# Behavioural plasticity of *Anopheles coluzzii* and *Anopheles arabiensis* undermines LLIN community protective effect in a Sudanese-savannah village in Burkina Faso

**DOI:** 10.1186/s13071-020-04142-x

**Published:** 2020-06-01

**Authors:** Eleonora Perugini, Wamdaogo Moussa Guelbeogo, Maria Calzetta, Sara Manzi, Chiara Virgillito, Beniamino Caputo, Verena Pichler, Hilary Ranson, N’Fale Sagnon, Alessandra della Torre, Marco Pombi

**Affiliations:** 1 Dipartimento di Sanità Pubblica e Malattie Infettive, Laboratory affiliated to Istituto Pasteur Italia, Fondazione Cenci Bolognetti, Sapienza Università di Roma, Rome, 00185 Italy; 2 Centre National de Recherche et Formation sur le Paludisme (CNRFP), Ouagadougou 01, BP 2208 Burkina Faso; 3 Dipartimento di Biodiversità ed Ecologia Molecolare, Centro Ricerca e Innovazione, Fondazione Edmund Mach, San Michele all’Adige, Trento, Italy; 4 Department of Vector Biology, Liverpool School of Tropical Medicine, Liverpool, L3 5QA UK

**Keywords:** *Anopheles*, Malaria transmission, Sporozoite rate, Vector behaviour, Bednet

## Abstract

**Background:**

Despite the overall major impact of long-lasting insecticide treated nets (LLINs) in eliciting individual and collective protection to malaria infections, some sub-Saharan countries, including Burkina Faso, still carry a disproportionately high share of the global malaria burden. This study aims to analyse the possible entomological bases of LLIN limited impact, focusing on a LLIN-protected village in the Plateau Central region of Burkina Faso.

**Methods:**

Human landing catches (HLCs) were carried out in 2015 for 12 nights both indoors and outdoors at different time windows during the highest biting activity phase for *Anopheles gambiae* (*s.l.*). Collected specimens were morphologically and molecularly identified and processed for *Plasmodium* detection and L1014F insecticide-resistance allele genotyping.

**Results:**

Almost 2000 unfed *An. gambiae* (*s.l.*) (54% *Anopheles coluzzii* and 44% *Anopheles arabiensis*) females landing on human volunteers were collected, corresponding to a median number of 23.5 females/person/hour. No significant differences were observed in median numbers of mosquitoes collected indoors and outdoors, nor between sporozoite rates in *An. coluzzii* (6.1%) and *An. arabiensis* (5.5%). The estimated median hourly entomological inoculation rate (EIR) on volunteers was 1.4 infective bites/person/hour. Results do not show evidence of the biting peak during night hours typical for *An. gambiae* (*s.l.*) in the absence of bednet protection. The frequency of the L1014F resistant allele (*n* = 285) was 66% in *An. coluzzii* and 38% in *An. arabiensis*.

**Conclusions:**

The observed biting rate and sporozoite rates are in line with the literature data available for *An. gambiae* (*s.l.*) in the same geographical area before LLIN implementation and highlight high levels of malaria transmission in the study village. Homogeneous biting rate throughout the night and lack of preference for indoor-biting activity, suggest the capacity of both *An. coluzzii* and *An. arabiensis* to adjust their host-seeking behaviour to bite humans despite bednet protection, accounting for the maintenance of high rates of mosquito infectivity and malaria transmission. These results, despite being limited to a local situation in Burkina Faso, represent a paradigmatic example of how high densities and behavioural plasticity in the vector populations may contribute to explaining the limited impact of LLINs on malaria transmission in holo-endemic Sudanese savannah areas in West Africa.
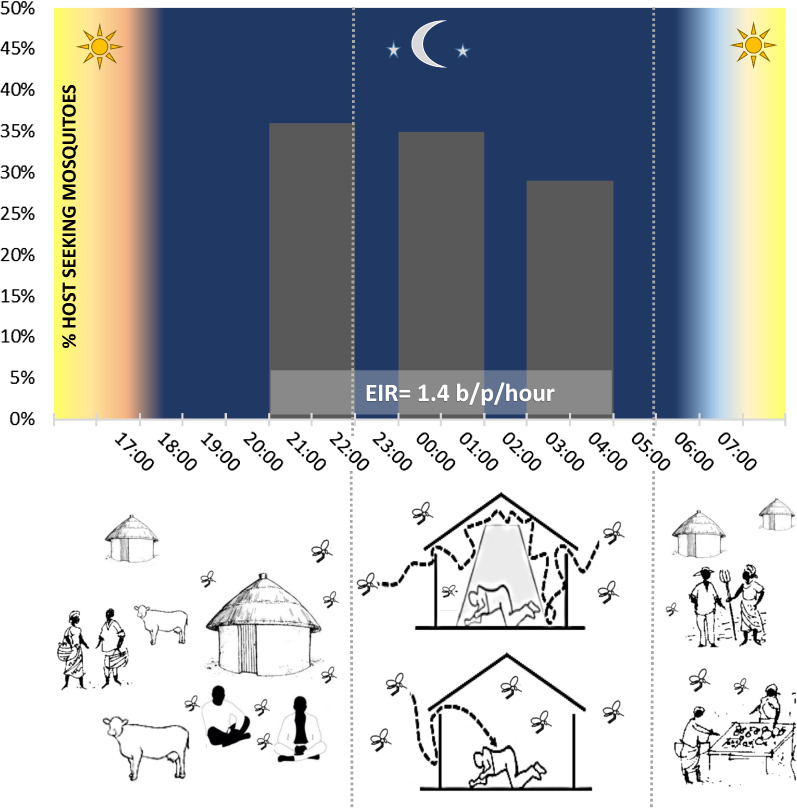

## Background

Long-lasting insecticide treated nets (LLINs) are very effective in reducing malaria transmission by combining individual physical protection to people sleeping under the nets with collective protection provided by the insecticidal activity of pyrethroids restrained in the net fibres [1]. Published data show that this community effect is reached when net usage in the population exceeds 50% [2, 3]. Since 2005, massive campaigns of three-year periodical distributions of LLINs have been implemented in many malaria-endemic countries. In sub-Saharan African countries, about 254 million LLINs were supplied between 2008–2010 and a further 806 million between 2011–2016 [1, 4]. It has been estimated that 68% of the 663 million malaria cases prevented in the first 15 years of this century in Africa, are due to the usage of LLINs [5]. Despite this success, most of sub-Saharan Africa still carries a disproportionately high share of the global malaria deaths [6–9] and the WHO has registered a stalling of the progress in the fighting against malaria since 2015. Particularly, the effectiveness of LLINs seems to be heterogeneous in some sub-Saharan hyper-endemic countries where the annual incidence is still very high [10].

Among many causal factors for this scenario (e.g. bednet quality and usage, ecological context), a crucial role is played by insecticide resistance, which undermines LLINs collective protective effect by reducing mosquito exposure to lethal dose of pyrethroids within the nets. Indeed, multiple mechanisms of resistance to pyrethroid insecticides have been observed in African *Anopheles* populations [11–13]: increased metabolic detoxification of insecticide molecules by enhanced enzyme activity (e.g. P450 monooxygenase) [13], mutations in insecticide target site (e.g. mutations in the para-type sodium channel gene, *kdr* mutations [14]) and/or mosquito behavioural adaptations (e.g. increased exophily, opportunistic biting activity and/or shift in biting activity [15]). Moreover, according to Killeen et al. [16], vector species composition could be a limiting factor to reduce mosquito density to a level sufficient to obtain a relevant impact on malaria transmission through LLINs: massive abatement can be expected where major vector species feed mostly indoors on humans. On the other hand, where generalistic/opportunistic species are dominant, LLINs need to be combined with other interventions to obtain an effect on vector population and subsequently on malaria transmission.

This might be the case for Burkina Faso where the increase in LLIN coverage, from 20% to 70% between 2009–2014, did not significantly affect malaria annual incidence, with an increasing number of cases reported each year [5, 17–23]. In a previous study carried out in 2011 in the village of Goden (Burkina Faso) one year after LLINs introduction, we observed an unexpected high sporozoite rate (SR) in the major malaria vectors in the area (i.e. 7.6% in *Anopheles coluzzii* and 5.3% in *Anopheles arabiensis*) despite low human blood index (20.1% in *An. coluzzii* and 5.8% in *An. arabiensis* [24]). Similar infective rates were also confirmed in a subsequent entomological survey conducted in the same village in 2012 (SR = 6.6% in *An. coluzzii* [25]). These observations suggest that, despite LLINs having significantly reduced human/vector contact in holo-endemic areas such as Burkina Faso, they have not apparently led to a substantial reduction of mosquito infection rates.

Thanks to human landing catches carried out in the same village five years after mass LLIN introduction, we here provide evidence on how high densities and behavioural plasticity of *An. coluzzii* and *An. arabiensis*, in association with insecticide resistance mechanisms, undermine the community protective effect of LLIN and reduce the impact of LLINs on malaria transmission in the Ziniaré district of Burkina Faso.

## Methods

### Sampling area

Field collections were carried out in November 2015 (i.e. at the end of rainy season, just after the peak of malaria transmission) in a holo-endemic [26] Sudanese-savannah village in the Plateau Central region (12° 25′ N, 1° 21′ W; Zinaré health district), 41 km East of Ouagadougou, the capital city of Burkina Faso. The sampling occurred in Goden, a rural village with approximately 800 inhabitants mainly belonging to the Mossi ethnic group, mostly devoted to agriculture, and rearing a few animals and occasional settlements of Fulani ethnic group devoted to cattle. From 2010 LLINs were widely distributed in the Plateau Central region. Although specific data on the actual LLIN coverage, quality and usage in the study site are not available, more than 9 million LLIN have been distributed in the whole country during 2013 campaign [27], with 64% of population coverage estimated in the Plateau Central region [28], a level above the threshold needed to elicit a community protective effect [2, 3].

### Entomological collections and molecular analysis

Host-seeking mosquitoes were collected by human landing catch (HLC) in two houses, both indoors and outdoors, at three different time windows (21:00–22:00 h; 00:00–01:00 h; 03:00–04:00 h) for a total of 12 nights. This interval corresponds to the highest biting activity phase reported for *An. gambiae* (*s.l.*) [29]. During the sampling period, no other human host was present in the houses with the exception of the volunteer who performed the collection and no LLINs or IRS were used.

All mosquitoes were morphologically identified under stereomicroscope [30], separated by species and sex. Heads and thoraces of *An. gambiae* (*s.l.*) females were dissected from abdomens and stored individually in tubes containing desiccant. DNA was extracted from heads and thoraces according to DNAZOL protocol (Molecular Research Center, Cincinnati, Ohio) [31]. Species were molecularly identified by SINE-PCR [32]. DNA from heads and thoraces was used as templates for *Plasmodium* sporozoite DNA detection by real-time PCR [33]. Subsamples of *An. arabiensis* and *An. coluzzii* were further processed by real-time PCR for genotyping L1014 (*kdr-w*), the most ancient and common insecticide-resistance associated allele in the sodium-gated voltage-channel gene [14, 34, 35].

### Statistical analysis

Differences in biting activity indoors *vs* outdoors and among different time windows of HLC collections were analysed by Mann-Whitney and Kruskal-Wallis tests (after assessment of non-normal distribution of data by Shapiro-Wilk test) for *An. gambiae* (*s.l.*), as well as for single species of the complex. Chi-square test was employed to investigate possible differences in sporozoite rate among species and between positions of sampling within each species. Chi-square test was also applied to assess prospective diversities in insecticide resistance level between species, between indoor *vs* outdoor collections within each species and between different infectivity states.

Generalized linear mixed effect models (GLMM) were built to verify variation in the abundance of vector species (*Anopheles gambiae* (*s.l.*), *An. arabiensis*, *An. coluzzii*) between trapping positions (indoors, outdoors), between houses (A and B) and among different HLC time windows (21:00–22:00 h; 00:00–01:00 h; 03:00–04:00 h). As the response variable of mosquito abundances is highly over-dispersed, a negative binomial distribution was chosen. For each species, two models were built, both including time windows, trapping locations and houses as covariates and the sampling days as a random effect. The two models differed in: (i) no interaction among variables; and (ii) interaction between trapping location and house. The best model was chosen by the Akaike information criterion (AIC) and likelihood ratio test. The tests were conducted using the R statistical software version 3.5.0 [36] with *lme4* package [37].

Overall hourly entomological inoculation rate (EIR) was calculated multiplying the median number of human biting mosquitoes in an hour (obtained by HLC data) with the estimated sporozoite rate.

## Results

During the 12 nights of sampling, 1996 unfed mosquito females were collected landing on human volunteers, corresponding to a median number of 23.5 females/hour/person. All of them were morphologically identified as *An. gambiae* (*s.l.*), of which 53.9% were *An. coluzzii*, 43.5% *An. arabiensis* and 0.5% *An. gambiae* (*s.s*). One *An. coluzzii*/*An. gambiae* (*s.s*) hybrid was found and 42 specimens (2.1%) were not successfully identified by PCR (Table 1).

**Table 1:** Percentage of mosquito abundance divided per species, time window and position of collection (indoors and outdoors)

Species	Indoors				Outdoors				Total (*n*)
	21:00–22:00 h	00:00–01:00 h	03:00–04:00 h	Total	21:00–22:00 h	00:00–01:00 h	03:00–04:00 h	Total	
*An. arabiensis*	24	41	35	422	35	36	30	446	868
*An. coluzzii*	34	39	27	519	46	26	28	556	1075
*An. gambiae*	100	0	0	5	0	40	60	5	10
*An. coluzzii/An. gambiae*	0	100	0	1	0	0	0	0	1
Unidentified	37	37	26	19	57	26	17	23	42
Total (*n*)	289	385	292	966	426	309	295	1030	1996

**Fig. 1:**
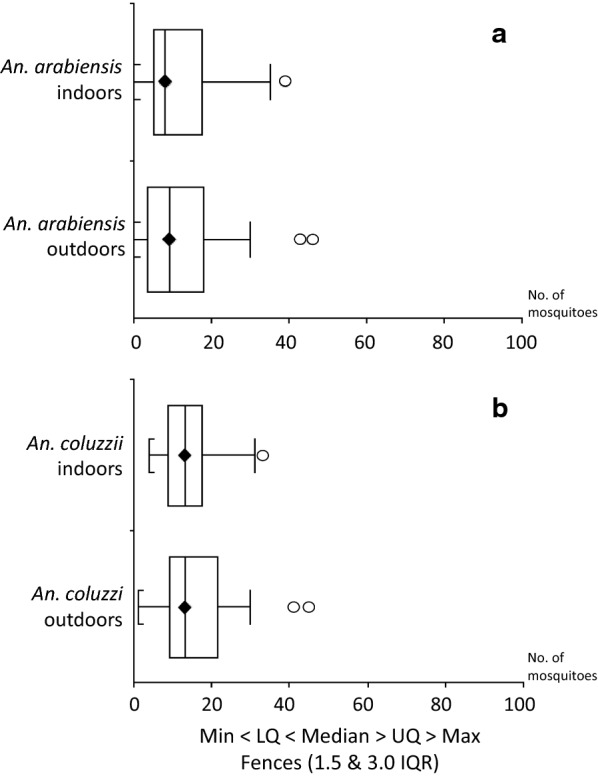
Box-and-Whisker plots of host-seeking females/hour/person (x-axis) collected indoors and outdoors. **a***Anopheles arabiensis*. **b***Anopheles coluzzii*. *Abbreviations*: min, minimum value; lq, lower quartile; uq, upper quartile; max, maximum value

No significant differences were observed in median numbers of mosquitoes collected indoors and outdoors (Mann-Whitney test: *An. arabiensis*, *U* = 0.51, *P* = 0.90; *An. coluzzii*, *U* = 0.52, *P* = 0.77; *An. gambiae* complex, *U* = 0.52, *P* = 0.74; Fig. 1). GLMM results indicated a higher abundance of *An. arabiensis* and *An. coluzzii* outdoors in one of the two houses sampled (see Additional file 1: Table S2). No significant differences were observed in median numbers of females collected during the three HLC-time windows (Kruskal-Wallis test; GLMM results: *An. arabiensis*, T = 1.16, *P* = 0.56; *An. coluzzii*, T = 2.84, *P* = 0.24; *An. gambiae* complex, T = 0.83, *P* = 0.66; Additional file 1: Table S2).

Overall, a 5.8% sporozoite rate (SR) was estimated: 114 *An. gambiae* (*s.l.*) females were found positive for *P. falciparum*, 1 for *Plasmodium* sp. (either *P. vivax*, *P. ovale* or *P. malariae*), and 1 for mixed infection (i.e. presence of both *P. falciparum* and *P. vivax/P. ovale/P. malariae*). No significant differences in SR were detected between *An. coluzzii* (6.1%) and *An. arabiensis* (5.5%) (*χ*^2^ = 0.2, *P* = 0.7), nor between samples collected indoors and outdoors (*An. arabiensis*: 6.6% indoors, 4.9% outdoors, *χ*^2^ = 1.5, *P* = 0.22; *An. coluzzii* 6.9% indoors, 5.2% outdoors, *χ*^2^ = 1.1, *P* = 0.24). The estimated median hourly entomological inoculation rate (EIR) was 1.4 infective bites/hour per human volunteer, corresponding to a cumulative EIR of 9.8 infective bites during the 7-hour sampling interval.

The observed frequency of *kdr*-*w* resistant allele was 66% in *An. coluzzii* (*n* = 163) and 38% in *An. arabiensis* (*n* = 122). The frequency of the homozygous resistant genotype was significantly higher in *An. coluzzii* (*χ*^2^ = 59.4, *P* < 0.0001) (Table 2). No differences in resistance genotypes were detected in indoors *vs* outdoors collected samples (*An. coluzzii*, *χ*^2^ = 0.3, *P* = 0.65; *An. arabiensis*, *χ*^2^ = 1.4, *P* = 0.49), nor in infective (*n* = 100) *vs* not infective (*n* = 188) specimens (*χ*^2^ = 2.3, *P* = 0.32). This, in agreement with Traoré et al. [38] and Doumbe-Belisse et al. [39], does not confirm previous reports of higher *P. falciparum* infective status/susceptibility in *kdr-*resistant mosquitoes [40–42].

**Table 2:** Homozygote resistant (+/+), heterozygote (+/−), sensitive wild type (−/−) genotype frequencies (in %) of the L1014F mutation in subsampled *An. arabiensis* and *An. coluzzii*

Species	+/+	+/*−*	*−*/*−*	Total (*n*)
*An. arabiensis*	27.1	21.3	51.6	122
*An. coluzzii*	43.5	46.1	10.4	163
Total (*n*)	36.5	35.4	31.1	285

## Discussion

Our results show that at the end of rainy season, inhabitants of Goden are potentially exposed to at least ten infective bites/person/night, despite five years since the beginning of LLIN mass distribution campaign in Plateau Central region of Burkina Faso [28]. Indeed, the fraction of bednet-protected people (about two-thirds of the whole population [28]) are likely exposed to mosquito-bites in early evening and early morning, when not sleeping under the LLIN and receive much less infective bites than estimated in the present study. However, the high biting rates observed between 21:00 and 22:00 h (when most people are inside houses and possibly not yet protected by LLINs; CNRFP, unpublished data) suggest actual high levels of exposure of the Goden population. This levels of exposures are unusually high compared to EIRs reported (per night) before LLIN distribution in the same village (mean 7.4, min 2.5, max 17.0 [43]), as well as in the Plateau Central region of Burkina Faso (mean 2.3, min 0.2, max 8.3 [44, 45]), as well as those reported in other regions of Burkina Faso (2.4 [46]) and in other sub-Saharan African countries where LLINs are in use (ranging from 0.04 to 3.4 [47–63]).

The estimated EIR value is due to both high malaria vector densities (HBR = 23.5 females/hour/person) and very high levels of infectivity in the vector population (SR = 5.8%). Both biting rate and infectivity rate are in line with the few literature data available for *An. gambiae* (*s.l.*) in Goden (1.8 < SR < 12.1 [43]) and in the same geographical area before LLIN implementation (0.5 < HBR < 26.3 [64, 65]; 3 < SR < 10 [64–66] in 6 villages in a radius of 40 km from Ouagadougou) and comparable to SR in Goden in 2011 (6.9% [24]) and 2012 (6.6% [25]). Notably, even though SRs were assessed by different approaches in different studies, results by recently developed rDNA-based TaqMan assays [33, 67] do not significantly differ to those obtained by traditionally used CSP-ELISA [67].

Data also shed some light on other entomological factors, which could have reduced LLIN-effectiveness after 5-year implementation (i.e. endophagy *vs* exophagy, time of biting and genetic resistance to insecticides) in the two main malaria vector species in the village, i.e. *An. coluzzii* and *An. arabiensis*.

First, our results do not suggest endophagic preferences in either vector species, in agreement with what observed in *An. coluzzii* in other settings characterized by massive LLIN coverage (North-West Burkina Faso [46], Benin [68] and Bioko Island [69]). Although most studies focusing on resting mosquitoes indirectly suggest high endophagy for *An. coluzzii/An. gambiae*, the few studies carried out by HLC both indoors and outdoors show lack of preference for the biting location even before LLIN implementation [70–77]. Overall, this highlights that anthropophily is the main driver of the endophagic behaviour in *An. coluzzii*/*An. gambiae*. Consequently, the supposed higher exophagy of *An. arabiensis* is a consequence of its generalistic host preference [78, 79]. Thus, in cases of reduced human-host availability indoors due to LLIN, both *An. coluzzii* and *An. arabiensis* do not require a secondary adaptation to bite outdoors, as they are already adapted to do it. Notably, mathematical models suggest that even relatively modest changes in outdoor biting can have a substantial public health impact (e.g. a 10% increased outdoor biting activity could result in 10.6 million additional malaria cases in whole Africa, even assuming a 100% LLINs coverage) [29].

**Fig. 2:**
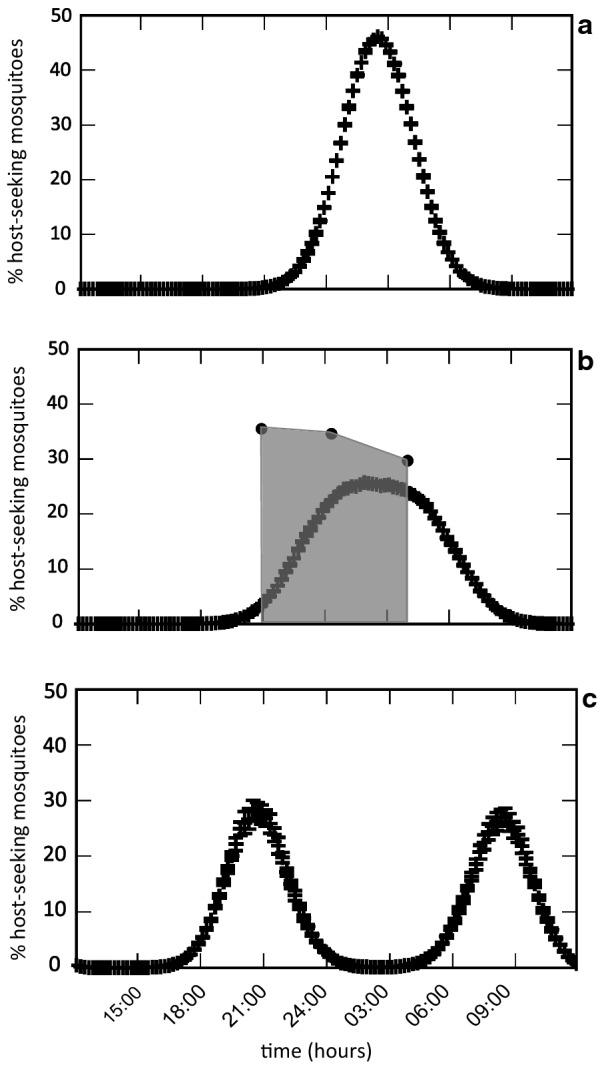
Predictive model describing the biting time profile of mosquitoes for different proportion of inhabitants using bednets: absence of coverage (a); intermediate coverage (**b**); full coverage (**c**). In **b** the model curve is overlapped with the human biting activity observed in sampling time windows in Goden, Burkina Faso (21:00–22:00 h; 00:00–01:00 h; 03:00–04:00 h). Differently from the theoretical model proposed by Ferreira et al. [90], in this study the curve of the biting activity is shrinked to three time windows, from which 100% of mosquitoes are counted. This results in a higher relative proportion of mosquitoes in this time window compared to the model curve (x-axis, hours; y-axis, percentage of biting mosquitoes). Modified from Ferreira et al. (2017) Modelling the impact of the long-term use of insecticide-treated bed nets on *Anopheles* mosquito biting time. Malar J. 16:373 [90] (Creative commons license http://creativecommons.org/licenses/by/4.0)

Secondly, no differences in biting rates were observed between 21:00 and 4:00 h in vector species. Indeed, a peak of activity during night hours is typical for *An. gambiae* (*s.l.*) in the absence of bednet protection [71, 72, 74, 75, 80–85]. To our knowledge, a lack of peak of activity was observed in *An. coluzzii* only in Burkina Faso [46] and in Bioko Island [69] where, two years after the introduction of LLINs, the proportion of host-seeking events changed towards a lack of significant differences throughout the night. On the other hand, a wide range of peak biting times (i.e. early, late or “central” night activity) has been reported in *An. arabiensis* after control interventions [52, 86–89]. According to theoretical predictions, lack of biting time peak is indicative of a situation in which the selective pressure exerted by LLIN has altered the typical biting pattern, but has not been yet sufficient to trigger a strong shift towards earlier and later biting times (Fig. 2; [90]) Expanding the duration of HLC before dusk and after dawn would allow to analyse more in detail a possible peak of biting activities to earliest and latest hours to access hosts unprotected by LLINs, as reported by Russell et al. [91]. In particular, measuring the numbers of host-seeking mosquitoes at times when people are still engaged in working activities outdoors would give a better estimate of actual risk of malaria transmission in the area.

Thirdly, while we did not carry out insecticide resistance bioassays, we genotyped a subsample of collected specimens for the locus L1014 of the sodium-gated voltage-channel gene, known to be one of the markers associated to pyrethroid resistance, to speculate on the possible role of insecticide resistance in contributing to high levels of transmission in the village by increasing survival of endophagic vectors entering in contact with the bednet. We found frequencies of the L1014F allele of 67% and 38% in *An. coluzzii* and *An. arabiensis*, respectively (without significant differences between indoor and outdoor collections), suggesting that pyrethroids are selecting target site resistance in the study site. Notably, the deterrent effect of LLINs on partially resistant (either behaviourally or genetically) mosquitoes is known to diverge biting activity to unprotected hosts both indoors and outdoors [92–95].

Effectiveness of ITN/LLINs relies on anthropophilic and therefore endophagic behaviour of vector populations and susceptibility to insecticides used to impregnate the bednets. A limited number of studies showed that changes in vector biting behaviour, as well as shift in species dominance, following LLIN implementation can undermine the efficacy of such control measures (Additional file 1: Table S3). A species shift was repeatedly shown in East Africa where, after LLIN introduction, the former most abundant highly anthropophilic vector, *An. gambiae*, has been outnumbered by the more generalist *An. arabiensis*, leading to an overall decrease in sporozoite rates [51–53, 88, 96–100]. To our knowledge, shifts in vector dominance after LLIN introduction has never been investigated in West and Central Africa (where *An. coluzzii* is also present), with the exception of Bioko Island, where a progressive replacement of *An. gambiae* by *An. coluzzii* was observed as indoor control measures were applied [69]. Our results do not show a shift in species composition following 5-year LLIN implementation in the study area in Burkina Faso where, even before bednet introduction, *An. coluzzii* and *An. arabiensis* were the main circulating vectors and *An. gambiae* was uncommon [101]. Nevertheless, our results suggest that LLINs have elicited a “behavioural resilience” (*sensu* Govella et al. [102]) in *An. coluzzii*, leading to the biting rhythms observed in the present study [72, 75, 84], as well as to a higher zoophagy, as detected in 2011 [24]. Indeed, *An. coluzzii* is known to be characterized by high ecological plasticity in the exploitation of different habitats [103–109], as well as by an opportunistic host-seeking behaviour [16, 69, 110]. Overall, the ecological plasticity of the two main vectors maximises their capacity to reach the human host when not protected by bednets, consistent with the high biting and sporozoite rates observed in the study site.

## Conclusions

Our results, despite being limited to a local situation in Burkina Faso, represent a paradigmatic example of how behavioural plasticity in the vector population may contribute to explain the limited impact of LLINs on malaria transmission in malaria holo-endemic Sudanese savannah areas in West Africa. Data suggest that the capacity of the two main vectors in the study site (*An. coluzzii* and *An. arabiensis*) to adjust their host-seeking behaviour to bite humans despite bednet protection, coupled with high densities and insecticide resistance, can undermine LLIN community protective effect, allowing the maintenance of high rates of mosquito infectivity and malaria transmission. The behavioural plasticity of *An. coluzzii* here highlighted suggests that this species is capable to react to indoor control interventions as shown in the case of *An. arabiensis* in East Africa. This should not be neglected when modelling the efficacy or planning malaria control measures at the local/regional level.

## Supplementary information

**Additional file 1.** : **Table S1.** Generalized linear mixed models built to evaluate the role of sampling conditions in explaining distributions of species abundance in study. In all models a negative binomial distribution has been chosen for the response variable, including time window, position and house as fixed effect and date as random effect. AIC: Akaike Information Criterion; d.f.: degrees of freedom; χ^2^: chi-square test value. **Table S2.** Summary of GLMM chosen for each species tested. **Figure S1.** Boxplots showing differences in abundances of *Anopheles gambiae* (*s.l.*), *Anopheles arabiensis* and *Anopheles coluzzii* according to the house and sampling position. **Table S3.** Changes in biting behaviour (time, exophagy and zoophagy), species dominance and sporozoite rate of mosquitoes of the *A. gambiae* species complex after LLIN/ITN introduction in sub-Saharan countries.

## Data Availability

Data supporting the conclusions of this article are included within the article and its additional files. The datasets used and analyzed during the present study are available from the corresponding author upon reasonable request.

## Abbreviations

CNRFP: Centre National de Recherche et de Formation sur le Paludisme
EIR: entomological inoculation rate
HLC: human landing catch
LLIN: long-lasting insecticide treated net
SR: sporozoite rate
