# Supplementary material for: Behavioural plasticity of Anopheles coluzzii and Anopheles arabiensis undermines LLIN community protective effect in a Sudanese-savannah village in Burkina Faso
Source: Parasit Vectors. 2020 Jun 1;13:277. doi: 10.1186/s13071-020-04142-x (PMC7268364; doi:10.1186/s13071-020-04142-x)
Supplement: Supplementary file 1 — Additional file 1. : Table S1. Generalized linear mixed models built to evaluate the role of sampling conditions in explaining distributions of species abundance in study. In all models a negative binomial distribution has been chosen for the response variable, including time window, position and house as fixed effect and date as random effect. AIC: Akaike Information Criterion; d.f.: degrees of freedom; χ2: chi-square test value. Table S2. Summary of GLMM chosen for each species tested. Figure S1. Boxplots showing differences in abundances of Anopheles gambiae (s.l.), Anopheles arabiensis and Anopheles coluzzii according to the house and sampling position. Table S3. Changes in biting behaviour (time, exophagy and zoophagy), species dominance and sporozoite rate of mosquitoes of the A. gambiae species complex after LLIN/ITN introduction in sub-Saharan countries. [file 13071_2020_4142_MOESM1_ESM.docx]

**Additional file 1**

**Text S1. Statistical analysis describing the effect of sampling conditions in variations describing mosquito abundances.**

**Methods**

Generalized linear mixed effect models (GLMM) were built within the statistical software R version 3.5.0 (2018-04-23) augmented with lme4 packages for statistical analysis. The analysis was conducted to verify variation in the abundance of vector species (*Anopheles gambiae* (*s.l*.), *Anopheles arabiensis, Anopheles coluzzii*) between trapping positions (indoors, outdoors), between houses (house A and B) and among different time windows (21:00-22:00 h; 00:00-01:00 h; 03:00-04:00 h). The response variable of mosquito abundances were highly over-dispersed, so they were modelled using a negative binomial distribution. For each species, two models were built, including time point, trapping location, house as covariates, sampling day as random effect and: i) no interaction among variables ii) interaction between trapping location and house. The two models were compared using the Akaike information criterion (AIC) and likelihood ratio test.

**Results**

The mean abundance of *An. gambiae* (*s.l.*)*,* *An. arabiensis*, *An. coluzzii* was best explained by a model that included the interaction between trapping position and house (Tables S1 and S2) indicating a statistically significant higher abundance of mosquitoes outdoors in house B compared for both *An. arabiensis* and *An. coluzzii* (Figure S1).

**Table S1.**  [Generalized linear mixed models built to evaluate the role of sampling conditions in explaining distributions of species abundance](https://static-content.springer.com/esm/art%3A10.1186%2Fs12936-019-3030-5/MediaObjects/12936_2019_3030_MOESM3_ESM.docx) in study. In all models a negative binomial distribution has been chosen for the response variable, including time point, position and house as fixed effect and date as random effect. AIC: Akaike Information Criterion; d.f.: degrees of freedom; χ^2^: chi-square test value.

| **Species tested** | **Model** | **AIC** | **d.f.** | **χ^2^** | **p-value** |
| --- | --- | --- | --- | --- | --- |
| *Anopheles gambiae* (*s.l.*) | no interaction | 650 | 1 | 20.73 | <0.005 |
|  | position*house | 669 |  |  |  |
| *Anopheles arabiensis* | no interaction | 465 | 1 | 15.03 | 0.0001 |
|  | position*house | 478 |  |  |  |
| *Anopheles coluzzii* | no interaction | 493 | 1 | 19.61 | <0.005 |
|  | position*house | 510 |  |  |  |

**Table S2**. Summary of GLMM chosen for each species tested.

| **Species tested** | **Parameter** | **Estimate** | **Standard error** | **z-value** | **p-value** |
| --- | --- | --- | --- | --- | --- |
| *Anopheles*  *gambiae* (*s.l.*) | intercept | 3.94 | 0.17 | 21.90 | <0.0001 |
|  | time point 03:00-04:00 h | -0.22 | 0.15 | -1.600 | 0.11 |
|  | time point 21:00-22:00 h | 0.04 | 0.15 | 0.33 | 0.72 |
|  | position OUT | -0.54 | 0.17 | -3.30 | <0.005 |
|  | house B | -0.51 | 0.22 | -2.23 | <0.005 |
|  | position OUT:house B | 1.17 | 0.24 | 5.00 | <0.005 |
| *Anopheles*  *arabiensis* | intercept | 2.75 | 0.26 | 10.12 | <0.0001 |
|  | time point 03:00-04:00 h | -0.22 | 0.15 | -1.34 | 0.17 |
|  | time point 21:00-22:00 h | -0.22 | 0.16 | -1.24 | 0.21 |
|  | position OUT | -0.44 | 0.18 | -2.26 | 0.024 |
|  | house B | -0.72 | 0.36 | -1.97 | 0.05 |
|  | position OUT:house B | 1.11 | 0.28 | 4.11 | <0.0001 |
| *Anopheles*  *coluzzii* | intercept | 2.82 | 0.14 | 18.86 | <0.0001 |
|  | time point 03:00-04:00 h | -0.21 | 0.13 | -1.47 | 0.14 |
|  | time point 21:00-22:00 h | 0.17 | 0.14 | -1.34 | 0.18 |
|  | position OUT | -0.51 | 0.15 | -3.22 | 0.001 |
|  | house B | -0.39 | 0.18 | -2.10 | 0.034 |
|  | position OUT:house B | 1.09 | 0.23 | 4.81 | <0.0001 |

**Figure S1**. Boxplots showing differences in abundances of *Anopheles gambiae* (*s.l.*), *Anopheles arabiensis* and *Anopheles coluzzii* according to the house and sampling position.

**Table S3.** Changes in biting behaviour (time, exophagy and zoophagy), species dominance and sporozoite rate of mosquitoes of the *A. gambiae* species complex after LLIN/ITN introduction in sub-Saharan countries. N.A.: information not available; SR: sporozoite rate.

| Country | Area | Dominant species  pre-introduction | Dominant species post-introduction | Increased Exophagy | Biting Time | Increased Zoophagy | SR(%) pre-introduction | SR(%) post-introduction | References |
| --- | --- | --- | --- | --- | --- | --- | --- | --- | --- |
| Bioko Island | Punta Europa | *A. gambiae* s.s. | *A. coluzzii* | + | N.A. | N.A. | N.A. | N.A. | [1] |
| Senegal | Dielmo | *A. gambiae* s.l. *A. funestus* | *A.gambiae s.l. A.funestus (reduced density)* | + | no | N.A. | 1% | 2.2-0.5% | [2] |
| Kenya | Kenya coast | *A. gambiae* s.s. | *A. arabiensis* | + | + | + | 3.8-8.7% | 0.2-0.3% | [3–6] |
| Kenya | Asembo area | *A. gambiae* s.s. | *A. arabiensis* | + | + | N.A | 6-13% | 0.5-8.4% | [7] |
| Kenya | Asembo-Seme | *A. gambiae* s.s. | *A. arabiensis* | N.A. | N.A. | N.A | 6.9% | 1.6% | [8] |
| Kenya | Seme | *A. gambiae* s.s. | *A. arabiensis* | N.A. | N.A. | + | 6.9% | 2% | [8–10] |
| Kenya | Kewale district | *A. gambiae* s.l. *A. funestus* | *A. gambiae* s.l.  *A. funestus* | N.A. | N.A. | + | N.A. | N.A | [11] |
| Tanzania | Kilombero Valley (Niaji, Lupiro) | *A. gambiae* s.s. | *A. arabiensis* | no | + | N.A | N.A. | N.A | [12] |
| Tanzania | Kilombero Valley (Idete, Namawala) | *A. gambiae* s.s. | *A. arabiensis* | N.A. | N.A. | N.A. | 2.5% | 0.3% | [13] |
| Tanzania | Usa River | N.A | N.A | N.A. | + | N.A | N.A. | N.A | [14] |
| Tanzania | Muheza area (Mng'aza, Milingano, Umba villages) | *A. gambiae* s.s. | *A. gambiae s.s. (reduced density)* | + (Milingano only) | + (Mng'aza only) | no | 5.3-5.1% | 1.3-2.3% | [15] |

**References**

1. Reddy MR, Overgaard HJ, Abaga S, Reddy VP, Caccone A, Kiszewski AE, et al. Outdoor host seeking behaviour of Anopheles gambiae mosquitoes following initiation of malaria vector control on Bioko Island, Equatorial Guinea. Malar J. BioMed Central Ltd; 2011;10:184.

2. Sougoufara S, Thiaw O, Cailleau A, Diagne N, Harry M, Bouganali C, et al. The impact of periodic distribution campaigns of long-lasting insecticidal-treated bed nets on malaria vector dynamics and human exposure in Dielmo, Senegal. Am J Trop Med Hyg. 2018;98:1343–52.

3. Mbogo CNM, Baya NM, Ofulla AVO, Githure JI, Snow RW. The impact of permethrin-impregnated bednets on malaria vectors of the Kenyan coast. Med Vet Entomol. 1996;10:251–9.

4. Mwangangi JM, Mbogo CM, Orindi BO, Muturi EJ, Midega JT, Nzovu J, et al. Shifts in malaria vector species composition and transmission dynamics along the Kenyan coast over the past 20 years. Malar J. 2013;12:1–9.

5. Mutuku FM, King CH, Mungai P, Mbogo C, Mwangangi J, Muchiri EM, et al. Impact of insecticide-treated bed nets on malaria transmission indices on the south coast of Kenya. Malar J. BioMed Central Ltd; 2011;10:356.

6. Mbogo CM, Mwangangi JM, Nzovu JG, Gu W, Yan G, Gunter JT, et al. Spatial and temporal heterogeneity of Anopheles mosquitoes and Plasmodium falciparum transmission along the Kenyan coast. Am J Trop Med Hyg. 2003;68:734–42.

7. Bayoh MN, Walker ED, Kosgei J, Ombok M, Olang GB, Githeko AK, et al. Persistently high estimates of late night, indoor exposure to malaria vectors despite high coverage of insecticide treated nets. Parasites and Vectors. 2014;7:1–13.

8. Lindblade KA, Gimnig JE, Kamau L, Hawley WA, Odhiambo F, Olang G, et al. Impact of Sustained Use of Insecticide-Treated Bednets on Malaria Vector Species Distribution and Culicine Mosquitoes. J Med Entomol. 2006;43:428–32.

9. Bayoh MN, Mathias DK, Odiere MR, Mutuku FM, Kamau L, Gimnig JE, et al. Anopheles gambiae: Historical population decline associated with regional distribution of insecticide-treated bed nets in western Nyanza Province, Kenya. Malar J. 2010;9:1–12.

10. Joshi GP, Service MW, Pradhan GD. A survey of species A and B of the anopheles gambiae giles complex in the kisumu area of kenya prior to insecticidal spraying with OMS-43 (fenitrothion). Ann Trop Med Parasitol. 1975;69:91–104.

11. Bøgh C, Pedersen EM, Mukoko DA, Ouma JH. Permethrin-impregnated bednet effects on resting and feeding behaviour of lymphatic filariasis vector mosquitoes in Kenya. Med Vet Entomol. 1998;12.

12. Russell TL, Govella NJ, Azizi S, Drakeley CJ, Kachur SP, Killeen GF. Increased proportions of outdoor feeding among residual malaria vector populations following increased use of insecticide-treated nets in rural Tanzania. Malar J. 2011;10:1–10.

13. Russell TL, Lwetoijera DW, Maliti D, Chipwaza B, Kihonda J, Charlwood JD, et al. Impact of promoting longer-lasting insecticide treatment of bed nets upon malaria transmission in a rural Tanzanian setting with pre-existing high coverage of untreated nets. Malar J. 2010;9:1–14.

14. Njau RJA, Mosha FW, Nguma JFM. Field trials of pyrethroid impregnated bednets in northern Tanzania—1. Effect on malaria transmission. Int J Trop Insect Sci. 1993;14:575–84.

15. Magesa SM, Wilkes TJ, Mnzava AEP, Njunwa KJ, Myamba J, Kivuyo MDP, et al. Trial of pyrethroid impregnated bednets in an area of Tanzania holoendemic for malaria. Part 2. Effects on the malaria vector population. Acta Trop. 1991;49.
